# Supplementary material for: Canine Electroencephalography Electrode Positioning Using a Neuronavigation System
Source: Animals (Basel). 2024 May 23;14(11):1539. doi: 10.3390/ani14111539 (PMC11171057; doi:10.3390/ani14111539)
Supplement: Supplementary file 1 [file animals-14-01539-s001.zip › animals-3000824-supplementary.pdf]

Table S1 Breed, age, sex, weight, time and reason of death, death or elected euthanasia of dogs used in the study. M = male, Mc= male castrated, F= female, Fs= female spayed, eutha= euthanasia

Dog	Breed	Age (months)	Sex	Weight (kg)	Time of death (month-year)	Reason for euthanasia/ death	Euthanasia / natural death
1	French Bulldog	65	Mc	15	May-23	Myelopathy	eutha
2	Labradoodle	101	Fs	30	Nov-22	Neoplasia heart	eutha
3	Galgo español	37	Mc	26	Feb-23	Diaphragmatic hernia	eutha
4	Mixed breed	unknown	Fs	10	May-23	Unknown	death
5	German Shepherd Dog	23	M	28	Nov-22	SIRS, seizure	eutha
6	Mixed breed	66	M	32	Feb-23	Pneumothorax	death
7	Flat coated Retriever	101	Fs	28	Sep-22	Neoplasia kidney	eutha
8	Bearded Collie	101	Mc	21	Aug-22	Unknown	death
9	Mixed breed	28	M	35	Mar-23	Unknown	death
10	Mixed breed	182	Fs	25	Mar-23	Unknown	death
11	Shar Pei	89	M	21	Feb-23	Neoplasia spleen	eutha
12	Collie	21	M	20	Jun-22	Sepsis	unknown
13	German Shepherd Dog	53	Fs	34	Jul-22	Torsio ventriculi	eutha
14	Boxer	133	Fs	27	Jul-22	Seizures	eutha
15	Bernese Mountain Dog	96	Fs	34	Sep-22	Neoplasia urethra	eutha
16	Elo	160	Fs	23	Sep-22	Neoplasia	death
17	Labrador	115	F	33	Oct-22	Adrenal Tumor	eutha
18	Labrador	166	M	32	Nov-22	Cardiac disease	eutha
19	Bordeaux Mastiff	60	M	59	May-23	Cardiac disease	eutha
20	Whippet	195	F	10	May-23	Unknown	eutha
21	Fox Terrier	132	F	7	May-23	Neoplasia thorax	eutha
22	French Bulldog	12	M	12	May-23	Dyspnoe	death

Table S2 shows the alignment, potential deviation and in case of deviation the underlying brain structure / BPA, each individual electrode had for each dog. The alignment and/or deviation was compared to the BPA according to current literature for canine EEGs, which can be found in the second line of this table underneath each electrode pair. Each dogs breed, sex and weight ante mortem is listed.
Abbreviations: BPA = Brain projection area, AOI = alignment only in, Fp1 Fp2 = Left and right fronto-polar electrodes, F3 F4 = Left and right frontal electrodes, P3 P4 = Left and right parietal electrodes, O1 O2 = Left and right occipital electrodes, T3 T4 = Left and right temporal electrodes

		Fp1	Fp2	F3	F4	P3	P4	O1	O2	T3	T4
Desired brain projection area (BPA)		Frontal cortex: prorean gyrus		Agranular cortex: precruciate gyrus		Parietal cortex: rostral part of the ectomarginal gyrus		Granular cortex (occipital area): marginal/occipital gyrus		Granular cortex (temporal area): pseudosylvian fissure	
Dog 1 French Bulldog, male 15kg		Partial<br>lateral<br>precruciate/ prorean gyrus	Partial<br>lateral<br>precruciate/ prorean gyrus	Yes	Yes	No<br>lateral, caudal<br>medial ecto-marginal gyrus	No<br>lateral, caudal<br>medial ecto-marginal gyrus	Partial<br>caudal<br>AOI saggital plane	Partial<br>caudal<br>AOI saggital plane	No<br>caudal<br>Occipital Gyrus	No<br>caudal<br>Occipital Gyrus
Dog 2 Labradoodle, female, 30kg		Partial<br>lateral<br>AOI transverse plane	Partial<br>lateral<br>AOI transverse plane	Yes	Yes	No<br>lateral, caudal<br>medial ecto-marginal gyrus	No<br>lateral, caudal<br>medial ecto-marginal gyrus	Yes	Yes	No<br>caudal, dorsal<br>rostral ecto-sylvian gyrus	No<br>caudal, dorsal<br>rostral ecto-sylvian gyrus
Dog 3 Galgo Espanol, male, 26kg		Partial<br>lateral<br>AOI transverse plane	Partial<br>lateral<br>AOI transverse plane	Yes	Yes	Yes	Yes	Yes	Yes	No<br>caudal<br>caudal composite gyrus	Yes
Dog 4 Mixed breed, female, 10kg		Partial<br>lateral<br>AOI transverse plane	Partial<br>lateral<br>AOI transverse plane	No<br>lateral, caudal<br>Postcruciate gyrus	No<br>lateral, caudal<br>Postcruciate gyrus	No<br>lateral, caudal<br>medial ecto-marginal gyrus	No<br>lateral, caudal<br>medial ecto-marginal gyrus	Yes	Yes	No<br>caudal<br>caudal ecto-marginal gyrus	Yes
Dog 5 German Sheperd, male, 28kg	Alignment<br>Deviation<br>BPA	No<br>lateral, rostral<br>overlying no BPA	No<br>lateral, rostral<br>overlying no BPA	Yes	Yes	Yes	Yes	Yes	Yes	No<br>caudal<br>caudal ecto-marginal gyrus	Yes
Dog 6 Mixed breed, male, 35kg		Partial<br>lateral, rostral<br>AOI transverse plane	Partial<br>lateral, rostral<br>AOI transverse plane	Yes	Yes	No<br>lateral, caudal<br>caudal ecto-marginal gyrus	No<br>lateral, caudal<br>caudal ecto-marginal gyrus	Partial<br>lateral, caudal<br>AOI saggital plane	Partial<br>lateral, caudal<br>AOI saggital plane	No<br>caudal, dorsal<br>rostral ecto-sylvian gyrus	No<br>caudal, dorsal<br>rostral ecto-sylvian gyrus
Dog 7 Flat coated Retriever, female, 28kg		No<br>lateral, rostral<br>overlying no BPA	No<br>lateral, rostral<br>overlying no BPA	Partial<br>lateral<br>AOI transverse plane	Partial<br>lateral<br>AOI transverse plane	Yes	Yes	Partial<br>caudal<br>AOI saggital plane	Partial<br>caudal<br>AOI saggital plane	No<br>caudal, dorsal<br>rostral ecto-sylvian gyrus	No<br>caudal, dorsal<br>rostral ecto-sylvian gyrus
Dog 8 Bearded Collie, male, 21kg		No<br>lateral, rostral<br>overlying no BPA	No<br>lateral, rostral<br>overlying no BPA	No<br>lateral, rostral<br>overlying no BPA	No<br>lateral, rostral<br>overlying no BPA	No<br>lateral, rostral<br>precruciate gyrus	No<br>lateral, rostral<br>precruciate gyrus	No<br>rostral<br>medial ecto-marginal gyrus	No<br>rostral<br>medial ecto-marginal gyrus	No<br>dorsal<br>rostral ecto-sylvian gyrus	No<br>dorsal<br>rostral sylvian gyrus
Dog 9 Mixed breed, male, 35kg		No<br>lateral, rostral<br>overlying no BPA	No<br>lateral, rostral<br>overlying no BPA	Partial<br>lateral<br>AOI saggital plane	Partial<br>lateral<br>AOI saggital plane	Yes	Yes	Yes	Yes	No<br>caudal, dorsal<br>caudal ecto-marginal gyrus	No<br>caudal, dorsal<br>caudal ecto-marginal gyrus
		No	No	No	No	Yes	Yes	Yes	Yes	No	No

Dog 10<br>Mixed breed, female, 25kg		lateral, rostral<br>overlying no BPA	lateral, rostral<br>overlying no BPA	lateral, rostral<br>Prorean gyrus	lateral, rostral<br>Prorean gyrus					caudal, dorsal<br>rostral ecto-sylvian gyrus	caudal, dorsal<br>rostral ecto-sylvian gyrus
Dog 11<br>Shar Pei, male, 21kg		No<br>lateral, rostral<br>overlying no BPA	No<br>lateral, rostral<br>overlying no BPA	No<br>lateral, rostral<br>Prorean gyrus	No<br>lateral, rostral<br>Prorean gyrus	Yes	Yes	Yes	Yes	No<br>caudal, dorsal<br>medial ecto-sylvian gyrus	No<br>caudal, dorsal<br>rostral sylvian gyrus
Dog 12<br>Collie, male, 20kg		No<br>lateral, rostral<br>overlying no BPA	No<br>lateral, rostral<br>overlying no BPA	No<br>lateral, rostral<br>Prorean gyrus	No<br>lateral, rostral<br>Prorean gyrus	Yes	Yes	Yes	Yes	Yes	Yes
Dog 13<br>German Sheperd, female, 34kg		No<br>lateral<br>overlying no BPA	No<br>lateral<br>overlying no BPA	Yes	Yes	No<br>lateral, caudal<br>medial ecto-marginal gyrus	No<br>lateral, caudal<br>medial ecto-marginal gyrus	Yes	Yes	Yes	Yes
Dog 14<br>Boxer, female, 27kg		No<br>lateral, rostral<br>overlying no BPA	No<br>lateral, rostral<br>overlying no BPA	No<br>lateral, rostral<br>Prorean gyrus	No<br>lateral, rostral<br>Prorean gyrus	Yes	Yes	Yes	Yes	No<br>caudal<br>ectosylvian gyrus	No<br>caudal<br>caudal sylvian gyrus
Dog 15<br>Bernese Mountain Dog, female, 34kg	Alignment Deviation BPA	No<br>lateral, rostral<br>overlying no BPA	No<br>lateral, rostral<br>overlying no BPA	No<br>lateral, rostral<br>Prorean gyrus	No<br>lateral, rostral<br>Prorean gyrus	Yes	Yes	Yes	Yes	Yes	Yes
Dog 16<br>Elo, female, 23kg		No<br>lateral<br>overlying no BPA	No<br>lateral<br>overlying no BPA	Partial<br>lateral<br>AOI transverse plane	Partial<br>lateral<br>AOI transverse plane	Yes	Yes	Yes	Yes	No<br>caudal, dorsal<br>ectosylvian gyrus	No<br>caudal, dorsal<br>ectosylvian gyrus
Dog 17<br>Labrador, female 33kg		No<br>lateral, rostral<br>overlying no BPA	No<br>lateral, rostral<br>overlying no BPA	Partial<br>lateral, rostral<br>Prorean gyrus/ precruciate gyrus	Partial<br>lateral, rostral<br>Prorean gyrus/ precruciate gyrus	Yes	Yes	Yes	Yes	Yes	Yes
Dog 18<br>Labrador, male, 32kg		Partial<br>lateral<br>AOI transverse plane	Partial<br>lateral<br>AOI transverse plane	Yes	Yes	Yes	Yes	Yes	Yes	Yes	Yes
Dog 19<br>Bordeaux Mastiff, male, 59kg		No<br>lateral, rostral<br>overlying no BPA	No<br>lateral, rostral<br>overlying no BPA	Partial<br>lateral<br>AOI transverse plane	Partial<br>lateral<br>AOI transverse plane	No<br>lateral, caudal<br>medial ecto-marginal gyrus	No<br>lateral, caudal<br>medial ecto-marginal gyrus	Partial<br>lateral, caudal<br>AOI saggital plane	Partial<br>lateral, caudal<br>AOI saggital plane	No<br>caudal, dorsal<br>ectosylvian gyrus	No<br>caudal, dorsal<br>ectosylvian gyrus
Dog 20<br>Whippet, female, 10kg		Partial<br>lateral<br>AOI transverse plane	Partial<br>lateral<br>AOI transverse plane	Yes	Yes	Yes	Yes	Partial<br>caudal<br>AOI saggital plane	Partial<br>caudal<br>AOI saggital plane	No<br>caudal<br>ectosylvian gyrus	No<br>caudal<br>ectosylvian gyrus
Dog 21		Partial	Partial	No	No	Yes	Yes	Yes	Yes	Yes	Yes

Fox Terrier, female, 7kg		lateral<br>AOI transverse plane	lateral<br>AOI transverse plane	caudal<br>Postcruciate gyrus	caudal<br>Postcruciate gyrus						
Dog 22<br>French Bulldog, male, 12kg		Partial<br>lateral<br>precruciate/ prorean gyrus	Partial<br>lateral<br>precruciate/ prorean gyrus	Yes	Yes	Yes	Yes	Yes	Yes	No<br>caudal<br>Occipital Gyrus	No<br>caudal<br>Occipital Gyrus
